# Langerhans cell histiocytosis confined to extrahepatic bile duct causing sclerosing cholangitis in child: a case report

**DOI:** 10.1186/s40792-020-00899-6

**Published:** 2020-06-16

**Authors:** Masakazu Murakami, Shun Onishi, Yuki Ohya, Seiichi Kawabata, Kaori Isono, Yasuhiko Sugawara, Tsuguharu Asato, Yumi Honda, Yoshiki Mikami, Yukihiro Inomata, Taizo Hibi, Satoshi Ieiri

**Affiliations:** 1 Department of Pediatric Surgery, Research Field in Medicine and Health Sciences, Medical and Dental Sciences Area, Research and Education Assembly, Kagoshima University, 8-35-1, Sakuragaoka, Kagoshima City, 890-8520 Japan; 2 Department of Pediatric Surgery and Transplantation, Kumamoto University Graduate School of Medical Sciences, Kumamoto, Japan; 3 Department of Diagnostic Pathology, Kumamoto University Hospital, Kumamoto, Japan

**Keywords:** Langerhans cell histiocytosis, Sclerosing cholangitis, Biliary cirrhosis, Extrahepatic bile duct, Liver transplantation

## Abstract

**Background:**

Langerhans cell histiocytosis (LCH) is an abnormal accumulation of Langerhans cells in various organs that sometimes induces organ dysfunction. LCH can affect the liver, resulting in sclerosing cholangitis and biliary cirrhosis. However, liver and bile duct involvement is usually observed in the disseminated form of LCH. We herein report a rare case of LCH localized only in the extrahepatic bile duct that resulted in severe liver cirrhosis.

**Case presentation:**

A 3-year-old boy with elevated liver enzymes, obstructive jaundice, and dilation of the common bile duct was referred to our institution. Contrast-enhanced computed tomography showed atrophy of the right hepatic lobe, relative hypertrophy of the left hepatic lobe, choledocholiths, and biliary debris extensively with biliary duct dilation. Magnetic resonance cholangiopancreatography revealed dilation of the intrahepatic and extrahepatic bile ducts and multiple choleliths in the gallbladder and common bile duct. Laparoscopic cholecystectomy, intraoperative cholangiography, liver biopsy, and gastrointestinal fiberscopy were performed. A liver specimen showed severe biliary cirrhosis due to sclerosing cholangitis. The patient then underwent living-donor liver transplantation because of severe liver cirrhosis 3 months after the first surgery. The common bile duct was not suitable for duct-to-duct anastomosis and was resected because of severe inflammation. Histologic sections of the common bile duct showed histiocytic cell proliferation. Immunohistochemistry revealed histiocytoses that were positive for Langerin, S-100 protein, and CD1a. However, no histiocytic cell proliferation was noted in the liver tissue. The definitive diagnosis was LCH localized to the extrahepatic bile duct. LCH in the extrahepatic bile duct seemed to cause sclerosing cholangitis. The patient was discharged uneventfully 2 months after living-donor liver transplantation.

**Conclusions:**

LCH localized to the extrahepatic bile duct is extremely rare; however, LCH can still affect the extrahepatic bile ducts on occasion. LCH should be considered as a differential diagnosis if pediatric patients show the presence of sclerosing cholangitis.

## Background

Langerhans cell histiocytosis (LCH) is a wide spectrum of clinical disorders involving the abnormal accumulation of Langerhans cells in various organs [1]. Proliferation of abnormal Langerhans cells can result in organ dysfunction. LCH ranges from a self-healing solitary lesion to fetal multi-organ involvement [2]. It occasionally develops in the liver and is well known as a cause of sclerosing cholangitis and biliary cirrhosis in pediatric patients [3]. In such situations, LCH cells are usually detected in both the liver and intrahepatic bile duct [4].

We herein report a rare case of primary LCH localized to the extrahepatic bile duct inducing liver cirrhosis.

## Case presentation

A 3-year-old boy was referred to our institution for a definitive diagnostic workup because of elevated liver enzymes, obstructive jaundice, and dilation of the common bile duct. These findings were noted during the treatment of cholangitis at previous hospitals, and congenital biliary dilatation induced by pancreaticobiliary maljunction was suspected.

**Fig. 1:**
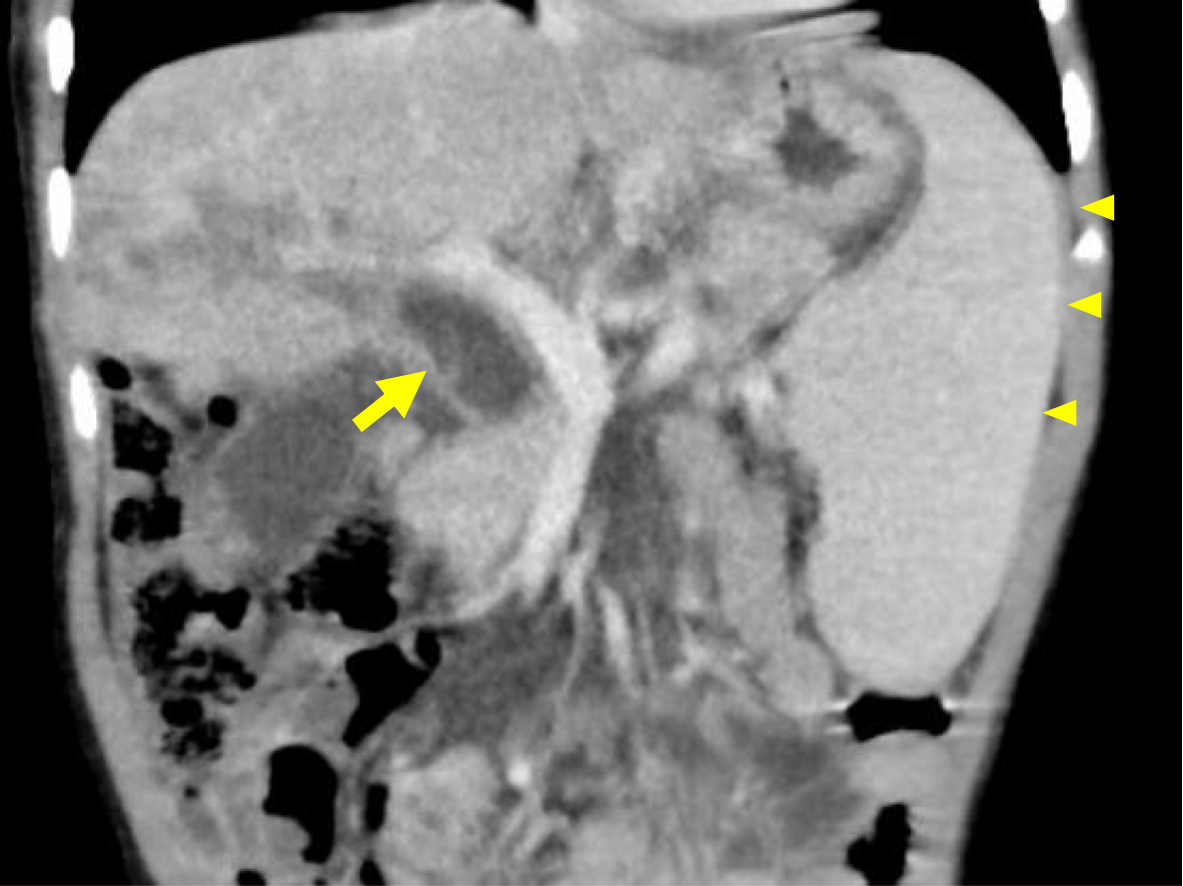
Contrast-enhanced computed tomography of the abdomen (coronal view). The common biliary duct was dilated (arrow). The right hepatic lobe was extremely atrophied, and splenomegaly was recognized (arrow head)

**Fig. 2:**
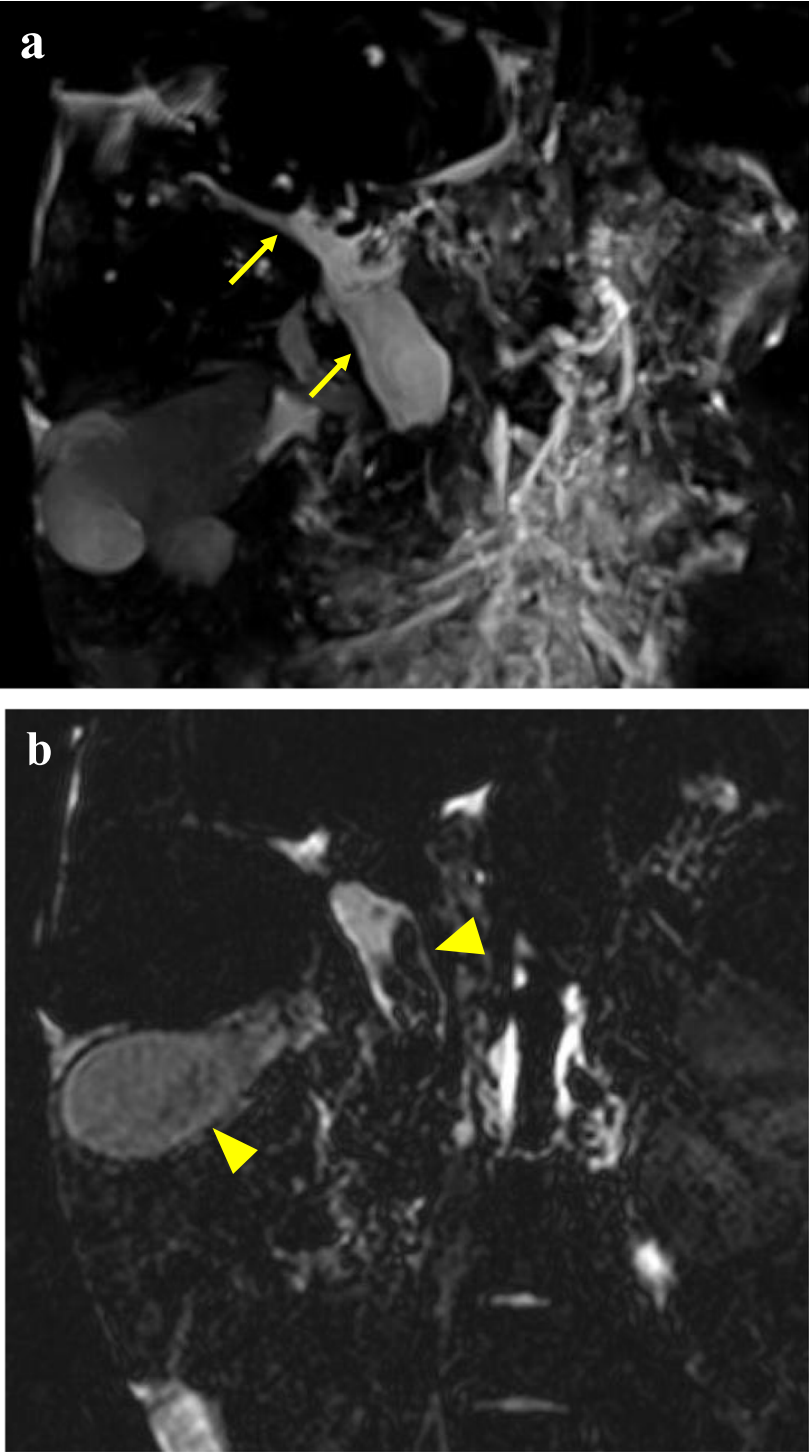
Magnetic resonance cholangiopancreatography (coronal view). **a** The intrahepatic and extrahepatic bile ducts were dilated (arrow). **b** Multiple choleliths were recognized in the gallbladder and common bile duct (arrowhead)

Laboratory data showed elevation of liver and biliary enzymes (aspartate aminotransferase 104 U/L, alanine aminotransferase 39 U/L, alkaline phosphatase 2206 U/L, γ-glutamyl transpeptidase 133 U/L, total bilirubin 1.7 mg/dL, and direct bilirubin 0.6 mg/dL). Contrast-enhanced computed tomography (CT) showed atrophic changes of the right hepatic lobe, enlargement of the left hepatic lobe, choledocholiths, and biliary debris with common bile duct dilation (Fig. 1). Enhanced CT also revealed splenomegaly and esophageal varix. Liver cirrhosis and portal hypertension were suspected based on these findings. Magnetic resonance cholangiopancreatography (MRCP) revealed dilation of the intrahepatic and extrahepatic bile ducts and multiple choleliths in the gallbladder and common bile duct (Fig. 2).

To obtain a definitive diagnosis, laparoscopic cholecystectomy, intraoperative cholangiography, liver biopsy, and gastrointestinal fiberscopy (GIF) were performed. Typical liver cirrhosis findings, such as atrophy of the right lobe and enlargement of the left lobe, were recognized by laparoscopic exploration. Intraoperative cholangiography revealed the normal junction of the main pancreatic duct and common bile duct, indicating no pancreaticobiliary maljunction. The intrahepatic bile duct of the right lobe was not visualized, and that of the left lobe was visualized irregularly. In addition, GIF revealed esophageal varices (Lm, F2, Cw, RC+). Laparoscopic cholecystectomy and liver biopsy were performed.

The pathological findings of the liver showed the disappearance of interlobular ductules, cholangiole proliferation, cholestasis, and extensive fibrosis. These findings were compatible with end-stage liver cirrhosis. Liver cirrhosis seemed to have been induced by sclerosing cholangitis, but the etiology of sclerosing cholangitis was unknown.

The postoperative course was uneventful, but the liver cirrhosis was progressive and in an end-stage condition. We concluded that the patient would require living-donor liver transplantation soon, so we referred him to another institution for living-donor liver transplantation.

**Fig. 3:**
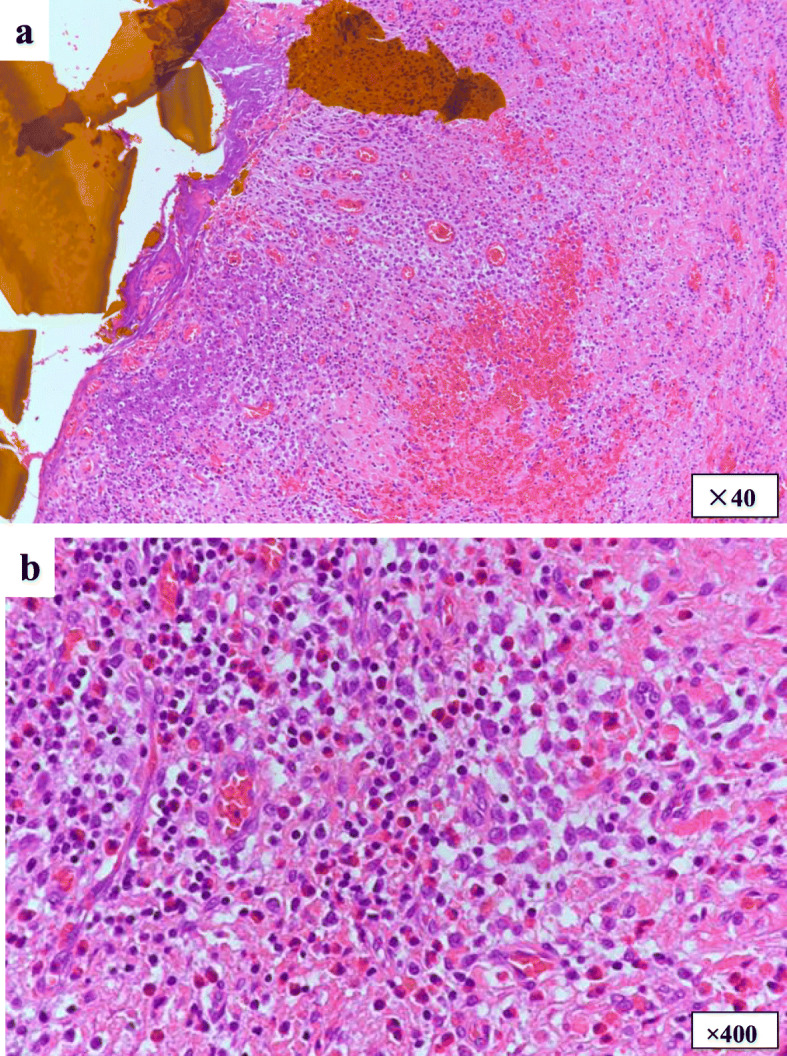
Pathological findings of the common bile duct. The submucosa of the common bile duct was diffusely infiltrated by a mixture of eosinophils and histiocytoid cells characterized by grooved and convoluted nuclei with scattered chromatin texture

**Fig. 4:**
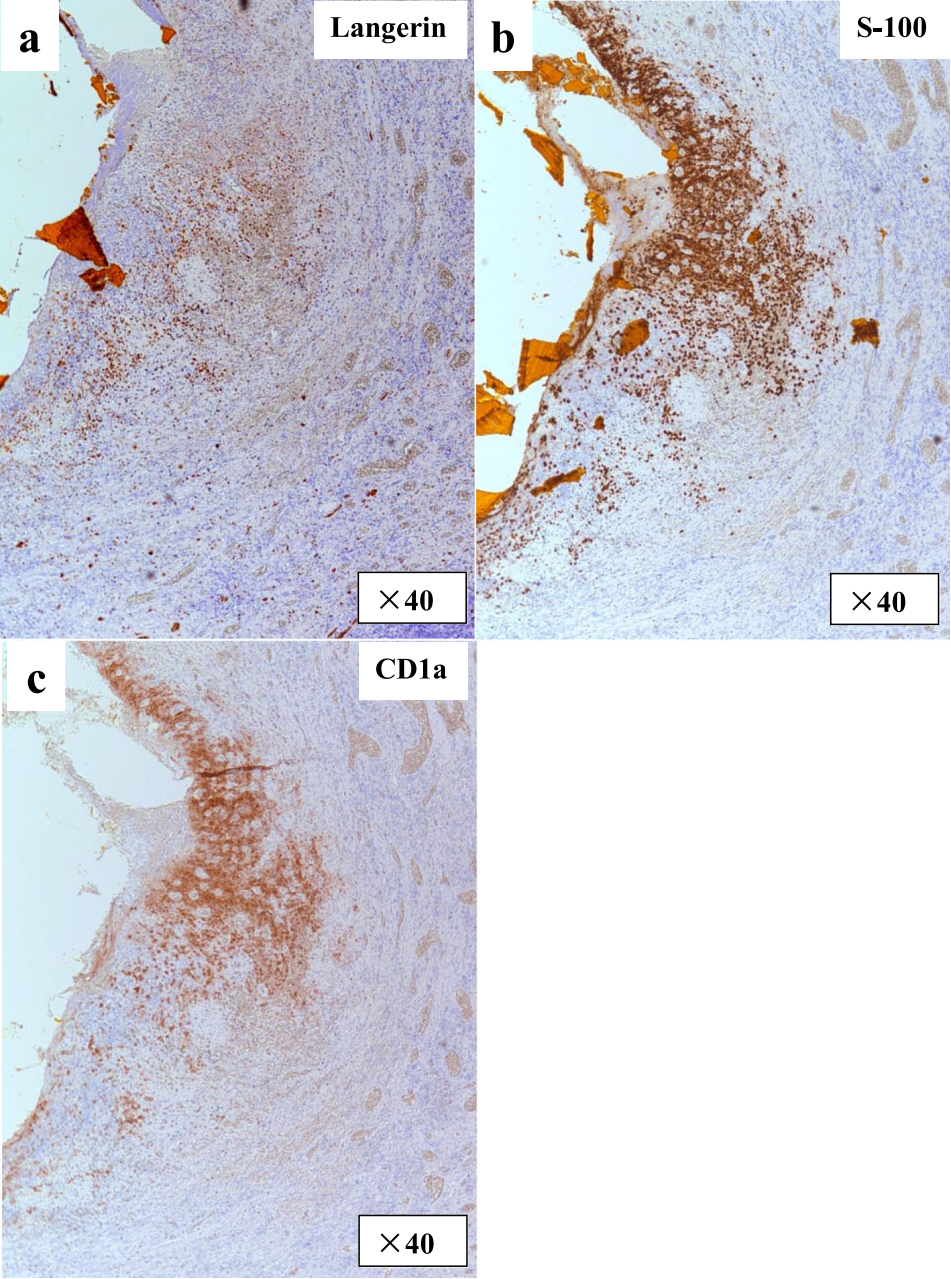
Immunohistochemical staining of the common bile duct. **a** Immunohistochemical staining of Langerin. **b** Immunohistochemical staining of S-100 protein. **c** Immunohistochemical staining of CD1a

The patient underwent living-donor liver transplantation from his father 3 months after the first surgery in our institution. The common bile duct had severe inflammation and was not suitable for duct-to-duct anastomosis. The hypertrophic common bile duct was resected, and the specimen was examined pathologically to confirm the definitive diagnosis. Microscopically, the wall of the common bile duct was thickened with fibrosis and was diffusely infiltrated by a mixture of eosinophils and medium to large histiocytoid cells with pale eosinophilic cytoplasm and grooved and convoluted nuclei showing scattered chromatin texture (Fig. 3). Immunohistochemical staining demonstrated that these histiocytoid cells were positive for Langerin (CD207) (Fig. 4a), S-100 protein (Fig. 4b), and CD1a (Fig. 4c), indicating the phenotype of Langerhans cells. This particular population of cells was not identified in the sections of the liver parenchyma. Based on these findings, the diagnosis of LCH, localized to the extrahepatic bile duct, was established. The preexisting liver tissue showed expansion of the portal tract with bridging fibrosis and cholangiolar proliferation as well as degeneration and regeneration of epithelium lining bile ducts, suggesting secondary sclerosing cholangitis due to obstructive process.

The postoperative course following living-donor liver transplantation was uneventful, and the patient was discharged on postoperative day 62. The patient has been followed for about 4 years after living-donor liver transplantation, and there is no sign of recurrence of LCH.

## Discussion

LCH is a rare disease marked by the proliferation of Langerhans cells. The incidence of LCH in children under 20 years of age is 2.9 cases per million per year, and LCH mainly occurs in children ≤ 4 years old [5]. A pathological examination is essential to confirm the definitive diagnosis of LCH. According to the WHO classification of tumors, if the histiocytes are positive for CD1a or Langerin on immunohistochemistry, a definitive diagnosis can be obtained [6].

The clinical manifestations of LCH vary from self-healing solitary lesions to fetal multi-organ involvement. LCH has shown an extremely excellent 2-year overall survival of 98.7% [5]. The mortality rate in patients with single-site LCH or without risk-organ involvement is estimated to be < 5% [3]. However, the mortality rate in children with multi-site LCH and risk-organ involvement, such as hematopoietic organ, liver, spleen, or lung, is reported to be 10–50% [3]. Although our case was one of single-site LCH, it caused severe liver cirrhosis. As a result, LCH localized to the common bile duct is considered to be risk-organ involvement.

It is suggested that LCH arises from bone marrow-derived immature myeloid dendritic cells rather than from epidermal Langerhans cells [1]. In pediatric patients with single-site LCH, the most common site is the bone (82%), followed by the skin (12%) [7]. Liver and bile duct involvement is usually observed in the disseminated form of LCH [4]. It is unclear why LCH was localized to the extrahepatic bile duct in our case. Primary LCH localized to the extrahepatic bile duct in children is rare, and only one case has been described. Finn and Jaffe reported a female patient with neonatal jaundice. The patient was diagnosed with primary LCH localized to the extrahepatic bile duct at 2.5 years old [8]. As in our case, the patient showed progressive destruction of the extrahepatic bile duct, leading to sclerosing cholangitis and eventually to secondary liver cirrhosis. The exact reason for the sclerosing change of the whole intrahepatic bile duct and liver cirrhosis is unknown. Regarding the possible reason for these specific findings, some inflammatory effect for the liver or ischemic influence for the bile duct might be considered.

Treatment of LCH should be planned according to the clinical presentation and the extent of organ involvement. Treatment can range from no treatment (observation) to surgical intervention and radiotherapy or chemotherapy. The standard optimal treatment of primary LCH of the extrahepatic bile duct has not been determined because of the limited number of cases. Eight reports have described performing living-donor liver transplantation in children with end-stage liver cirrhosis secondary to LCH-related sclerosing cholangitis, and the overall survival rate was 87% after living-donor liver transplantation with a mean follow-up of 3.4 years [9]. Living-donor liver transplantation was successfully performed in the patient described in a previous report [8] as well as in our case. Immunosuppressive agents after living-donor liver transplantation may be helpful in preventing recurrence of LCH [8]. In our case, LCH was a single lesion localized to the extrahepatic bile duct and was resected completely, and the patient had taken immunosuppressive agents after living-donor liver transplantation. Therefore, it is considered that recurrence is unlikely, and there is no indication of chemotherapy for preventing recurrence.

In our case, if an early definitive diagnosis of LCH had been obtained before the progression of sclerosing cholangitis, less invasive treatment, such as chemotherapy or hepatico-jejunostomy with common bile duct resection, may have been attempted. Because pathological examination is essential for the diagnosis of LCH, in infants with LCH localized to the extrahepatic bile duct, the resection of the extrahepatic bile duct is thought to be necessary for diagnosis. In our case, CT, MRCP, and intraoperative cholangiography could not suspect a neoplastic lesion of the extrahepatic bile duct; therefore, the resection of the extrahepatic bile duct for the diagnosis could not be performed. Diagnosis of LCH localized to the extrahepatic bile duct is considered challenging unless imaging examinations show a clear neoplastic lesion.

In children, secondary sclerosing cholangitis accounts for 30% of overall cases of sclerosing cholangitis, and secondary sclerosing cholangitis may be caused by LCH, congenital immunodeficiency, and cystic fibrosis [10]. LCH localized only to the extrahepatic bile duct is a rare phenomenon and challenging to diagnose. However, the presence of sclerosing cholangitis in children should raise suspicion of LCH [11].

## Conclusions

LCH localized to the extrahepatic bile duct is a rare condition. LCH should be considered as a differential diagnosis if pediatric patients show the presence of sclerosing cholangitis.

## Data Availability

The datasets supporting the conclusions of this article are included within the article.

## Abbreviations

LCH: Langerhans cell histiocytosis
CT: Computed tomography
MRCP: Magnetic resonance cholangiopancreatography
GIF: Gastrointestinal fiberscopy
CBD: Common bile duct

## References

[1] Harmon CM, Brown N (2015). Langerhans cell histiocytosis: a clinicopathologic review and molecular pathogenetic update. Arch Pathol Lab Med.

[2] Henter JI, Tondini C, Pritchard J (2004). Histiocyte disorders. Crit Rev Oncol Hematol.

[3] Morimoto A, Oh Y, Shioda Y, Kudo K, Imamura T (2014). Recent advances in Langerhans cell histiocytosis. Pediatr Int.

[4] Obiorah IE, Velasquez AH, Kallakury B, Özdemirli M (2018). Primary Langerhans cell histiocytosis of the extrahepatic bile duct occurring in an adult patient. Balkan Med J.

[5] Horibe K, Saito AM, Takimoto T (2013). Incidence and survival rates of hematological malignancies in Japanese children and adolescents (2006-2010): based on registry data from the Japanese Society of Pediatric Hematology. Int J Hematol.

[6] Jaffe R, Weiss LM, Facchetti F (2008). Tumours derived from Langerhans cells. WHO classification of tumours of haematopoietic and lymphoid tissues, 4th edn.

[7] Morimoto A, Ishida Y, Suzuki N (2010). Nationwide survey of single-system single site Langerhans cell histiocytosis in Japan. Pediatr Blood Cancer.

[8] Finn LS, Jaffe R (1997). Langerhans’ cell granuloma confined to the bile duct. Pediatr Pathol Lab Med.

[9] Hadzic N, Pritchard J, Webb D (2000). Recurrence of Langerhans cell histiocytosis in the graft after pediatric liver transplantation. Transplantation..

[10] Roberts EA (1999). Primary sclerosing cholangitis in children. J Gastroenterol Hepatol.

[11] Al Salloom AA, Almalki ST, Almana H, Burdelski M (2013). Diabetes insipidus and sclerosing cholangitis in a child may be a clue to the diagnosis of Langerhans’ cell histiocytosis: a case report. Int J Health Sci.

